# THE 4MS OF BEHAVIORAL HEALTH IN AN AGE-FRIENDLY HEALTH SYSTEM: TESTING AN EDUCATIONAL FRAMEWORK

**DOI:** 10.1093/geroni/igae098.2165

**Published:** 2024-12-31

**Authors:** Erin Emery-Tiburcio, Laura Porter, Siqi Wang

**Affiliations:** Rush University, Chicago, Illinois, United States; Cherokee Health Systems, Knoxville, Tennessee, United States; Rush University Medical Center, Chicago, Illinois, United States

## Abstract

The 4Ms framework of an Age-Friendly Health System (What Matters, Medication, Mentation, Mobility) was originally developed for hospital and ambulatory care settings. As many behavioral health providers did not clearly see a role for themselves in all 4Ms, the framework was adapted for use by behavioral health providers in order to improve the quality of care provided to older adults. The 4Ms-Behavioral Health (4Ms-BH) training program was designed and implemented to assess the impact of training on knowledge and clinical behavior change among mental health clinicians with little to no geriatric training. A secondary goal was to fine-tune the pilot training program for widespread implementation. Fifteen mental health clinicians from Community Mental Health Centers in three states completed eight hours of live webinar training over six months: one three-hour introduction followed by five monthly one-hour application webinars. Clinicians completed knowledge and clinical behavior measures before, during, and after training, along with an assessment of follow-up sustainability needs. The 4Ms-Behavioral Health framework was very well received by participants, who demonstrated significant knowledge gains and clinical behavior change. Participant knowledge increased for each of the 4Ms, with Medication and Mobility showing greatest improvement at 17% and 15%, respectively. Effect sizes for practice behavior change from pre-training to post-training were large (Cohen’s d range =.82 – 1.66, p≤.01) in each of the 4Ms assessment and action activities. Implications of the pilot training program for teaching students and licensed professionals will be discussed.

